# A potential adverse role for leptin and cardiac leptin receptor in the right ventricle in pulmonary arterial hypertension: effect of metformin is BMPR2 mutation-specific

**DOI:** 10.3389/fmed.2023.1276422

**Published:** 2023-10-05

**Authors:** Megha Talati, Evan Brittain, Vineet Agrawal, Niki Fortune, Katie Simon, Sheila Shay, Xiaofang Zeng, Michael L. Freeman, James West, Anna Hemnes

**Affiliations:** ^1^Division of Allergy, Pulmonary and Critical Care Medicine, Vanderbilt University Medical Center, Nashville, TN, United States; ^2^Division of Cardiovascular Medicine, Vanderbilt University Medical Center, Nashville, TN, United States; ^3^Department of Cardiology, Xiangya Hospital, Central South University, Changsha, China; ^4^Department of Radiation Oncology, Vanderbilt University Medical Center, Nashville, TN, United States

**Keywords:** pulmonary arterial hypertension, BMPR2 mutation, right ventricular dysfunction, RV lipotoxicity, leptin and leptin receptors, H9c2 cultured cardiomyocytes, mitochondrial respiration

## Abstract

**Introduction:**

Pulmonary arterial hypertension is a fatal cardiopulmonary disease. Leptin, a neuroendocrine hormone released by adipose tissue, has a complex relationship with cardiovascular diseases, including PAH. *Leptin is thought to* be an important factor linking *metabolic syndrome* and cardiovascular disorders. Given the published association between metabolic syndrome and RV dysfunction in PAH, we sought to determine the association between leptin and RV dysfunction. We hypothesized that in PAH-RV, leptin influences metabolic changes via leptin receptors, which can be manipulated by metformin.

**Methods:**

Plasma leptin was measured in PAH patients and healthy controls from a published trial of metformin in PAH. Leptin receptor localization was detected in RV from PAH patients, healthy controls, animal models of PH with RV dysfunction before and after metformin treatment, and cultured cardiomyocytes with two different BMPR2 mutants by performing immunohistochemical and cell fractionation studies. Functional studies were conducted in cultured cardiomyocytes to examine the role of leptin and metformin in lipid-driven mitochondrial respiration.

**Results:**

In human studies, we found that plasma leptin levels were higher in PAH patients and moderately correlated with higher BMI, but not in healthy controls. Circulating leptin levels were reduced by metformin treatment, and these findings were confirmed in an animal model of RV dysfunction. Leptin receptor expression was increased in PAH-RV cardiomyocytes. In animal models of RV dysfunction and cultured cardiomyocytes with BMPR2 mutation, we found increased expression and membrane localization of the leptin receptor. In cultured cardiomyocytes with BMPR2 mutation, leptin moderately influences palmitate uptake, possibly via CD36, in a mutation-specific manner. Furthermore, in cultured cardiomyocytes, the Seahorse XFe96 Extracellular Flux Analyzer and gene expression data indicate that leptin may not directly influence lipid-driven mitochondrial respiration in BMPR2 mutant cardiomyocytes. However, metformin alone or when supplemented with leptin can improve lipid-driven mitochondrial respiration in BMPR2 mutant cardiomyocytes. The effect of metformin on lipid-driven mitochondrial respiration in cardiomyocytes is BMPR2 mutation-specific.

**Conclusion:**

In PAH, increased circulating leptin can influence metabolic signaling in RV cardiomyocytes via the leptin receptor; in particular, it may alter lipid-dependent RV metabolism in combination with metformin in a mutation-specific manner and warrants further investigation.

## Introduction

Pulmonary arterial hypertension (PAH) is a rare but fatal disease characterized by increased muscularization of small arteries leading to elevated pulmonary arterial pressure, which ultimately leads to right ventricular (RV) dysfunction and failure. In PAH, lipotoxicity and impaired fatty acid oxidation are common features of the dysfunctional RV, and RV failure has been linked to metabolic disease (1). Leptin (Ob) is a 16 kDa non-glycosylated protein encoded by the obese (*ob*) gene and produced primarily by adipocytes (2). It is classically known as a key regulator of energy balance, including appetite (3, 4), though it can also have a wide range of effects that affect the cardiovascular, nervous, immune, and reproductive systems (5). In the left ventricle, leptin regulates cardiomyocyte contractility, apoptosis, and metabolism (6–9). Although leptin has the potential to alter metabolism in the RV and contribute to the metabolic phenotype of the failing RV, little is known about whether or how leptin may alter the RV in PAH.

Leptin may contribute to cardiovascular pathogenesis by altering fatty acid oxidation and cardiac hypertrophy, as well as increasing oxidative stress, vascular inflammation, endothelial dysfunction, and vascular proliferation (8, 10–12). Leptin mediates its effects by binding to leptin receptors (Ob-R), which are present in the heart, liver, kidney, brain, and pancreas. There are six isoforms of Ob-R (a–f) largely classified into three large groups: a long form (Ob-Rb), a short form (Ob-Ra, c, d, f), and a secretory form (Ob-Re). Ob-Rb is the dominant isoform in the heart, primarily responsible for leptin signaling and leptin resistance (10, 13, 14). In PAH, plasma leptin levels are increased (15, 16), and pulmonary endothelial cells (P-ECs) cultured from idiopathic PAH (IPAH) patients secrete more leptin than controls (15). In IPAH, leptin signaling contributes to regulatory T-cell dysfunction and may contribute to the development and progression of the PAH (17). In addition, in chronic hypoxia-induced rodent models of pulmonary hypertension (PH), abnormal activation of the Ob/Ob-Rb axis in the pulmonary vascular wall is required for increased leptin secretion by P-ECs and increased Ob-Rb expression in pulmonary artery smooth muscle cells (PASMCs) for PASMC proliferation (18, 19). In contrast, leptin-deficient mice are shown to recapitulate the histological features of PAH in the lung and heart (20), and lower leptin levels, when adjusted by BMI, are associated with increased overall mortality (21). These data show that the role of leptin is not well understood in the pulmonary vasculature, as there are conflicting data on its effects. Importantly, all these animal studies focused on the effect of the Ob/Ob-Rb axis specifically on pulmonary vascular cells but not on cardiomyocytes, which are responsible for RV dysfunction. The role of leptin in the RV is thus far understudied but may be relevant given the capacity of leptin to regulate organ-specific metabolism (17, 19).

In an open-label, phase II trial of metformin, an antidiabetic drug, in PAH patients, we have shown that metformin therapy is associated with improved RV function and, in a subset of patients, reduced RV triglyceride content that correlates with improved lipid and glucose metabolism markers (1). Furthermore, we and others have also shown that in animal models of RV load stress, metformin can improve RV function (22, 23). Prior publications have demonstrated a reduction in plasma leptin levels and liver steatosis with metformin administration (24). We, therefore, hypothesized that metformin administration in PAH may reduce elevated plasma leptin levels and sought to explore a role for leptin in the PAH-RV. Given the multiple metabolic derangements that occur in the PAH-RV including increased glycolysis and reduced fatty acid oxidation (25), it is possible that multiple metabolic interventions may be required to have maximal improvement in the failing RV. We, therefore, sought (1) to determine if metformin alters leptin abundance in the plasma in our human metformin trial and rodent models of PAH, suggesting that it is modifiable; (2) to test the role of leptin in regulating metabolism in PAH in our two animal models of RV load stress; and (3) to test for synergism in cardiomyocyte lipid-stimulated metabolism with multiple metabolic interventions.

## Methods

### Human blood samples

Fasting blood samples before and after metformin were collected as part of a pilot study of metformin in PAH that has been previously published, including patient demographics (1) (NCT01884051). Control and PAH fasting plasma were also collected as part of the Vanderbilt Prospective Pulmonary Hypertension Registry (26) and used for analyses. In each study, before study procedures, subjects provided written informed consent (VUMC IRB #9401, 140,608).

### H9c2-cultured cardiomyocyte cell line for leptin stimulation

H9c2 (ATCC CRL-1446), a rat (*Rattus norvegicus*) myoblast adherent cell line, was purchased from the American Type Culture Collection (ATCC, Manassas, VA, United States), and maintained in a growth medium comprising supplemented Dulbecco’s modified Eagle’s medium (DMEM), 10% fetal bovine serum (FBS), 2 mM glutamine, 1 mM pyruvate, 100 U/mL penicillin, and 100 mg/mL streptomycin in humidified air (5% CO_2_) at 37°C. To induce the differentiation into cardiac myocytes, the H9c2 myoblasts were transferred to a differentiating medium, which was composed of DMEM, 1% FBS, 2 mM glutamine, 1 mM pyruvate, 100 U/mL penicillin, and 100 mg/mL streptomycin. Cells starved in a differentiation medium for 48 h were used for experiments (27). These H9c2 cells were stably transfected with mutant BMPR2 plasmids [mutant 1 (M1), BMPR2 gene with a 2579-2580delT resulting in a frameshift at amino acid 859 resulting in 10 missense amino acids and a stop, and mutant 2 (M2), BMPR2 gene with a C993T mutation resulting in R332X], or empty vector (control) was used (28). G418 was used for the selection of positive clones. H9c2 cells from passages 8 to 12 were used for experiments. H9c2 cardiomyocytes were maintained in Dulbecco’s modified Eagle’s medium (DMEM) supplemented with 10% fetal bovine serum (FBS), 4.5 g/L d-glucose, 2 mM glutamine, 1 mM pyruvate, 100 U/mL penicillin, and 100 mg/mL streptomycin in humidified air (5% CO_2_) at 37°C. To induce the differentiation into cardiac myocytes, the H9c2 myoblasts were transferred to a differentiating medium, which was composed of DMEM, 1% FBS, 2 mM glutamine, 1 mM pyruvate, 100 U/mL penicillin, and 100 mg/mL streptomycin. Cells starved in a differentiation medium for 48 h were used for experiments. All the H9c2 clones expressed cardiac-specific markers (Acta1; Acta2; desmin; troponin T2, cardiac type; tropomyosin 1α) and demonstrated immunoreactivity for cardiac troponin T, markers of classical adult cardiomyocyte phenotype (27). For leptin stimulation, H9c2 cells in differentiation media were stimulated with 6 ng/mL Leptin (PeproTech, Cranbury, NJ) for 1 or 24 h.

### Measurement of H9c2 mitochondrial bioenergetics using the Seahorse XFe96 Extracellular Flux Analyzer

An XFe96 analyzer (Seahorse Biosciences, North Billerica, MA) was used to measure bioenergetic function in intact H9c2 cells in real time (29). Briefly, H9c2 cells were seeded at a density of 20,000 cells/well in Seahorse Bioscience XFe96 cell culture plates in 100 μL of cardiomyocyte differentiation media. The plates were incubated in a 37°C humidified incubator with 5% CO_2_ for 24 h to reach 70%–80% confluency. The next day, the cells were incubated for 2 h in cell starvation media (pH 7.4), followed by pretreatment with 6 ng/mL leptin or 6 ng/mL leptin +5 mM metformin for 1 h in fatty acid oxidation media (pH 7.4). Palmitate (100 μL) was added just before starting the assay. Normalization of the total number of nuclei per well following the experiment was used to control for variation in cell number. On completion of the assay, the cells were stained with Coomassie blue, and cell nuclei were counted using a cell counter. Oxygen consumption rate (OCR) data are expressed as pmol/min.

### Western blotting protocol for H9c2 cells

H9c2 cells were homogenized in RIPA buffer (PBS, 1% Ipegal, 0.5% sodium deoxycholate, and 0.1% SDS) with proteinase and phosphatase inhibitor cocktails (Sigma-Aldrich, St. Louis, MO). Protein concentration was determined by the Bradford assay (Pierce, Rockford, IL) and stored at −70°C until use. Primary antibodies used for Western blot included CD36 [3313; Cell Signaling Technologies (CSTs), Danvers, MA], Leptin Ab, and Na-ATPase, cell membrane-specific. Donkey anti-rabbit (711-035-152; Jackson ImmunoResearch Laboratories, West Grove, PA) was used as the secondary antibody.

### Animal studies

All animal procedures were approved by the Institutional Animal Care and Use Committee of the Vanderbilt University School of Medicine. We used a mouse model of mutant BMPR2 expression (30): the Rosa26-rtTA2 Å ~TetO7-BMPR2^R899X^ FVB/N mice previously described (31, 32), called BMPR2^R899X^ for brevity (33), in which mutant BMPR2 is universally expressed. Expression of transgene occurs only after the initiation of doxycycline. Transgene-negative mice were used as littermate controls and were administered doxycycline as well. BMPR2^R899X^ mice were fed a high-fat chow (60% lard, western diet) with 1 g/kg doxycycline beginning at 6 weeks of age. Western diet has been shown to increase the penetrance of PH in this model (34). This diet was continued for 6 weeks, at which time mice were euthanized and tissue was harvested.

### Pulmonary arterial banding mice for metformin study

Male C57/Bl6 mice were fed a WD consisting of 60% lard for 8 weeks (*n*¼16), beginning at 3 weeks of age, and weighed weekly. A subset of mice underwent pulmonary artery banding (PAB) as previously described (35) at 11 weeks of age and remained in place for 2 weeks, after which animals were sacrificed, with tissue harvested and preserved in formalin. Treated mice were given 2.5 g/kg metformin mixed in food (Bioserv, San Diego, CA, United States) beginning at 3 weeks of age and continuing until sacrifice at 13 weeks.

### Real-time polymerase chain reaction

RNA was isolated from the H9c2 cells using the Qiagen RNeasy Maxi Kit (Qiagen, Valencia, CA, United States), and first-strand complementary DNA (cDNA) was made using the QuantiTect^®^ Reverse Transcription Kit (Qiagen), both according to the manufacturer’s protocols. Quantitative real-time PCR analysis was performed using a total reaction volume of 25 μL, containing 5 μL of diluted cDNA, 12.5 μL SYBR Green Supermix (Applied Biosystems Foster City, CA, United States), and 0.03 μL of each oligonucleotide primer (250 mM). PCR was completed in the StepOnePlus Real-Time PCR System (Applied Biosystems) with HPRT as the housekeeping gene (Table 1).

**Table 1:** Primers sequence for genes used for RT-PCR in H9c2 cells.

Fasn_rat_651F	TTCAGGGAACGGGTATTGCC
Fasn_rat_771R	CTTGCAGCCATCTGTGTTCG
Acaca_rat_3600F	GCTCAACAGTGTACAGCATCG
Acaca_rat_3698R	GGGATGTTCCCTCTGTTTGG
Cpt1a_rat_860F	ACCGTGAGGAACTCAAACCC
Cpt1a_rat_1006R	CAACAATGTGCCTGCTGTCC
Cpt1b_rat_405F	TGGGTGGATGTTCGAGATGC
Cpt1b_rat_526R	GCTTGGGCAGTGATGTTTGG

### Immunofluorescence staining for leptin receptor in human and mouse paraffin-embedded RV tissue and H9c2 cells

Immunolocalization was performed on archival paraffin-embedded human RV tissue obtained from controls (*n* = 5; Cooperative Human Tissue Network) and HPAH patients (*n* = 4; Vanderbilt University Medical Center). The study protocol was approved by the Institutional Review Board of Vanderbilt University Medical Center (IRB 9401). Human and mouse RV tissue sections were deparaffinized, rehydrated, and blocked with 5% normal goat serum, followed by overnight incubation with leptin receptor antibody (1 μg/mL, Abcam, Waltham, MA) at 4°C. H9c2 cells were plated in four-well chamber slides (Catalog No.: 354104; Falcon) and grown until they reached 70% confluency and differentiated. Following stimulation with leptin (6 ng/mL; PeproTech, Cranbury, NJ), H9c2 cells were fixed with 4% paraformaldehyde for 15 min at 37°C and permeabilized (permeabilization buffer: 20 mM HEPES, pH 7.4, E8 300 mM sucrose, 50 mM NaCl, 3 mM MgCl_2_) for 10 min at room temperature, followed by incubation with leptin receptor antibody overnight at 4°C. The next day, RV tissue sections and H9c2 cells were incubated with Alexa Fluor 594-labeled secondary antibody (Sigma-Aldrich). The slides were mounted using Vectashield mounting media containing DAPI (Vector Laboratories, Burlingame, CA) for confocal microscopy. Semiquantitative analysis of immunofluorescence staining was conducted using NIS-Elements AR 4.11.00 64-bit software. All images were collected at the same time under the same conditions and were subjected to the same exposure time for images to be taken. The immunofluorescence intensity was calculated by dividing the sum of the immunofluorescence intensity measured per slice by the number of nuclei in that slice.

### Radioactive [^14^C] palmitate uptake by H9c2 cardiomyocytes in the presence of leptin

Palmitate uptake by H9c2 cardiomyocytes with and without mutant BMPR2 was quantified using the methods described by Stuck et al. (36) and Ha and Pak (37). Briefly, control, M1, and M2 cells were plated (2 × 10^4^ cells/well) in six-well plates in two sets of triplicates. After differentiation into cardiac myocytes, for a 24 h timepoint, the cells were incubated with or without leptin (6 ng/mL) for 24 h before adding radioactive palmitate. For incubation with leptin for a 1 h timepoint, the cells were incubated with leptin (6 ng/mL) along with 0.75 μCi/mL of [^14^C] palmitate for 60 min at 37°C. The uptake reaction was terminated by rapid washing with 1 mL of ice-cold PBS. Cells were disrupted with 1 mL of lysis buffer and scraping, and cell-associated radioactivity was determined by scintillation counting.

### Human and mouse plasma leptin ELISA assay

Human serum and mouse plasma samples were used for ELISA. Leptin ELISA for human samples was done using an Invitrogen ELISA kit (Cat. #KAC2281), and for mouse samples, it was done using an R&D Systems ELISA kit (Cat. #MOB00B). ELISA was performed following the manufacturer’s protocol.

### Statistical analysis

Statistical analyses for continuous variables were carried out using either one-way ANOVA, unpaired two-tailed *t*-tests, or Wilcoxon matched-pairs signed rank test (GraphPad Prism Software, La Jolla, CA). Data are expressed as means ± SE. *p* < 0.05 was considered significant.

## Results

### In PAH patients, plasma leptin levels are elevated, strongly correlate with BMI, and are reduced by metformin

**Figure 1:**
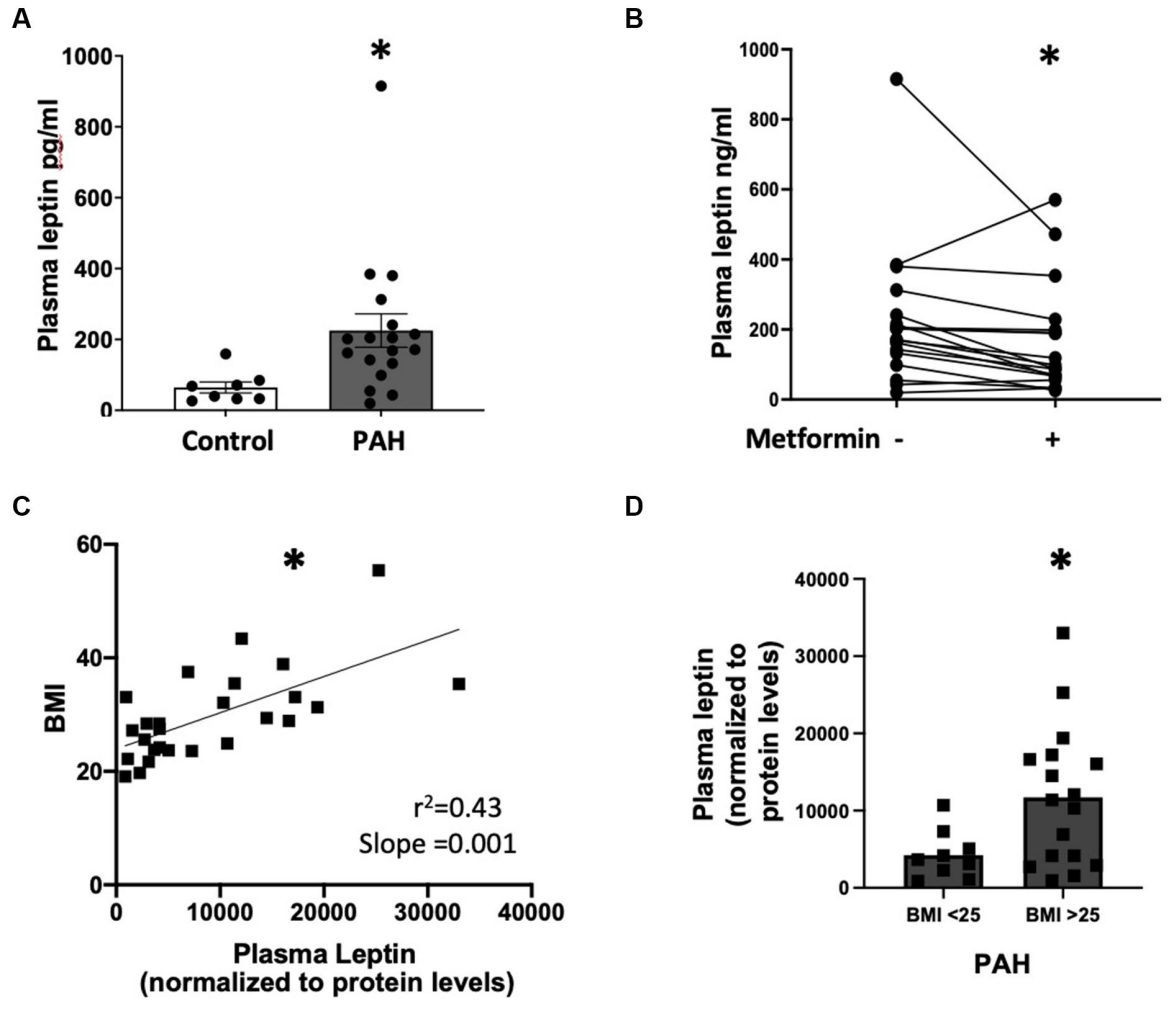
**(A)** Plasma leptin levels in controls (*n* = 8) and PAH (*n* = 18) patients ^*^p < 0.05. **(B)** Graph showing the change in plasma leptin from baseline after 8 weeks of metformin therapy for each patient (*n* = 18). ^*^p < 0.001. **(C)** Correlation between plasma leptin and BMI (*n* = 26) in PAH patients ^*^p < 0.001. **(D)** Plasma leptin in PAH patients with BMI <25 and BMI >25 ^*^p < 0.03. Samples used in **(A,B)** are from the metformin trial (NCT01884051). Samples used for **(C,D)** are from the Vanderbilt Prospective Pulmonary Hypertensive Registry (VUMC IRB #9401, 140608).

First, we observed that plasma leptin levels were significantly higher (*p* < 0.05) in PAH compared to healthy controls despite similar BMI (Figure 1A, BMI 29.11 ± 7.9 control vs. 29.38 ± 3.7 PAH, *p* = 0.9). In addition, plasma leptin levels were significantly reduced (*p* < 0.0001) following 8 weeks of metformin therapy (Figure 1B; Supplementary Figure S1A), despite a relatively modest weight loss of approximately 2 kg (1). Using the SomaLogic proteomic platform, we found that in PAH patients, leptin (9133.93 ± 8,245) was moderately correlated (*r* ^2^ = 0.43, slope = 0.001) (*p* < 0.001) with body mass index (BMI) (29.8 ± 8) (Figure 1C). Furthermore, when we stratified leptin based on BMI, PAH patients with BMI >25 had more than two-fold higher (*p* < 0.03) plasma leptin (11,719 ± 8,989) than patients with BMI <25 (4,251 ± 3,128) (Figure 1D). In addition, leptin showed a correlation with BMI in both BMI groups (Supplementary Figures S1B,C) (^*^p < 0.0001). Taken together, the data show that PAH patients have higher leptin levels in circulation than controls, despite having a similar BMI to controls, and that plasma leptin abundance correlates with higher BMI and is reduced by metformin treatment.

### In PAH-RV cardiomyocytes and animal models of RV load stress, leptin receptor expression is increased

**Figure 2:**
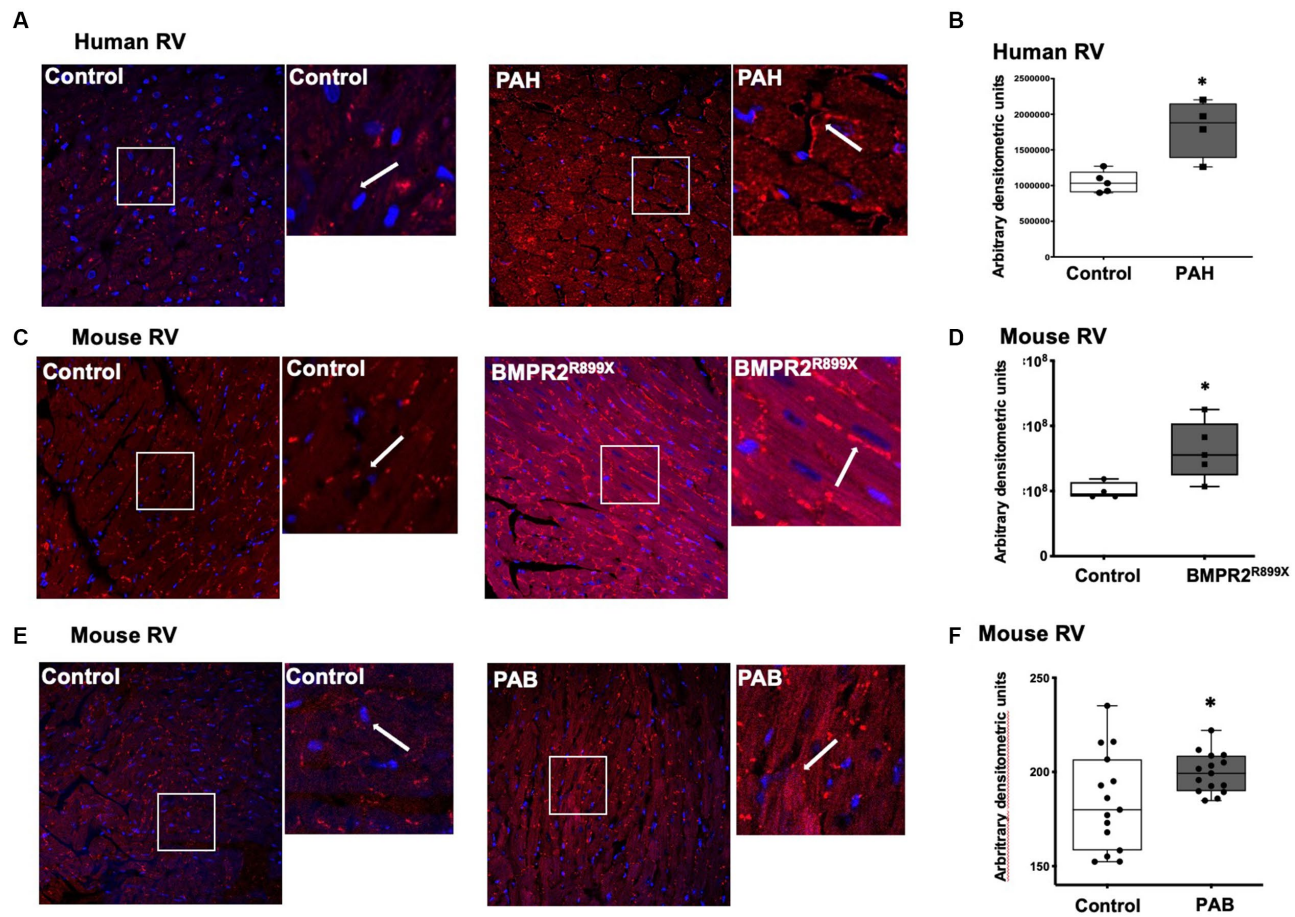
**(A)** Immunolocalization of leptin receptor in human RV from control (*n* = 5) and PAH (*n* = 4) patients. Magnification 400×. Magnified white box from the original picture on the right of control and PAH-RV tissue: leptin receptor immunolocalization in the cytoplasm and/or in the cell membrane (in red color), as indicated by the white arrow. **(B)** Bar graph showing leptin receptor staining intensity in control and PAH-RV tissue calculated using arbitrary densitometric units. **(C)** Immunolocalization of leptin receptor in RV tissue from control (*n* = 4) and BMPR2^R899X^ (*n* = 5) mice. Magnification 400×. Magnified white box from the original picture on the right of control and BMPR2^R899X^ mouse RV tissue: leptin receptor immunolocalization in the cytoplasm and/or in the cell membrane (in red color), as indicated by white arrow. **(D)** Bar graph showing leptin receptor staining intensity in control and BMPR2 mutant mouse RV tissue calculated using arbitrary densitometric units. **(E)** Immunolocalization of leptin receptor in RV tissue from control (*n* = 4) and PAB (*n* = 3). Magnification 400×. Magnified white box from the original picture on the right of control and PAB mouse RV tissue: leptin receptor immunolocalization in the cytoplasm and/or in the cell membrane (in red color), as indicated by white arrow. **(F)** Bar graph showing leptin receptor staining intensity in 4–5 random fields per mouse calculated using arbitrary densitometric units.

We next sought to determine if the PAH-RV has differences in the expression of leptin receptors, which are important in other forms of left heart failure (6, 7, 38–40) and may be targets for increased plasma leptin levels in PAH. In RV cardiomyocytes from human non-diseased controls, leptin receptor localization was mainly observed in the cytoplasm, whereas in RV cardiomyocytes from PAH patients, leptin receptor localization was observed both on the cell membrane (white arrow) and in the cytoplasm (Figure 2A). Semiquantitative analysis of leptin receptor staining indicated a significant increase (*p* < 0.01) in the expression of leptin receptors in PAH-RV cardiomyocytes (Figure 2B). We sought to recapitulate these findings using two rodent models of RV dysfunction: (1) mice with universal expression of BMPR2 mutation associated with heritable forms of PAH (BMPR2^R899X^), which we have previously shown to develop disproportional RV dysfunction to pulmonary vascular disease (35) and (2) pulmonary artery banding (PAB), a pure RV load-stress model. We found that in the RV cardiomyocytes from the BMPR2^R899X^ mutant mouse, both cytoplasmic and cell membrane localization of leptin receptor was strikingly increased (*p* < 0.05) compared to RV cardiomyocytes from the control mouse (Figures 2C,D). In PAB, we tested whether pure load stress could similarly alter the localization of leptin receptors in the RV. As shown in Figure 2E,F, the localization of the leptin receptor was significantly increased both in the cytoplasm and on the cell membrane in RV cardiomyocytes in PAB (*p* < 0.05) mice compared to control mice. Taken together, these data show a consistent increase in RV leptin receptor expression in humans with PAH and rodent models of RV dysfunction and stress.

### In a mouse model of RV dysfunction, metformin reduces plasma leptin and decreases the expression of leptin receptors in RV cardiomyocytes

**Figure 3:**
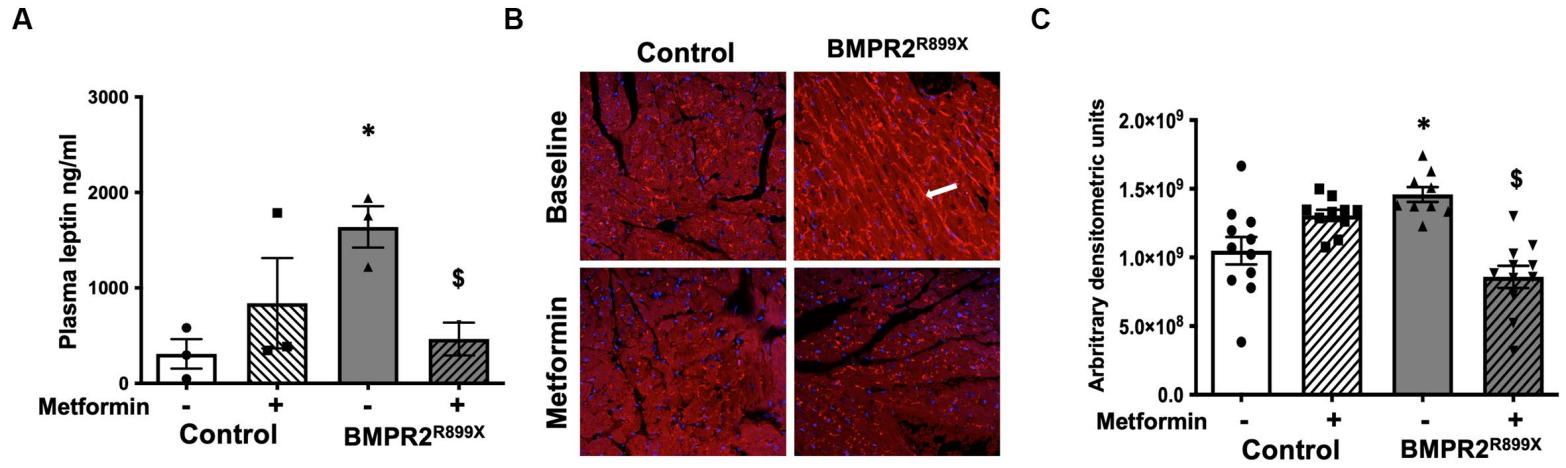
**(A)** Plasma leptin levels in control and BMPR2^R899X^ mice at baseline and following metformin treatment. ^*^p < 0.05 plasma leptin levels in control vs. BMPR2 mutant mice, ^$^*p* < 0.05 plasma leptin levels in BMPR2 mutant mice at baseline and following metformin treatment. **(B)** Immunolocalization of leptin receptor (red color immunofluorescence in the cytoplasm and cell membrane, as depicted by white arrow) in RV tissue from control (*n* = 4) and BMPR2^R899X^ (*n* = 5) mice at baseline and after metformin treatment. Magnification 200×. **(C)** Bar graph shows leptin receptor staining intensity in control and BMPR2^R899X^ mouse RV tissue at baseline and after metformin treatment calculated using arbitrary densitometric units. ^*^p < 0.05 protein expression in control vs. BMPR2 mutant mice, ^$^*p* < 0.05 protein expression in BMPR2 mutant mice at baseline and following metformin treatment. The statistical test used was a *t*-test.

In our human data, we found increased plasma leptin in PAH compared to controls, as well as increased RV leptin receptor abundance. We next sought to recapitulate these findings in a mouse model of RV dysfunction with BMPR2 mutation and test the hypothesis that reduced plasma leptin expression is accompanied by lower RV leptin receptors after metformin exposure. At baseline, plasma leptin levels were significantly increased (*p* < 0.01) in BMPR2^R899X^ mice compared to controls (Figure 3A). Following 6 weeks of metformin treatment, plasma leptin levels were significantly decreased (*p* < 0.05) in BMPR2^R899X^ mice compared to controls. In RVs from BMPR2^R899X^ mice, leptin receptor was also significantly increased (*p* < 0.01) at baseline compared to controls (Figures 3B,C). Following 6 weeks of metformin treatment, leptin receptor expression was significantly reduced (*p* < 0.0001) in BMPR2^R899X^ mice compared to controls (Figures 3B,C). These findings are consistent with our human data. We have previously shown a reduction in RV lipid content after metformin exposure in humans (1) and in RV dysfunction (22). These data indicate that metformin treatment leads to a reduction in plasma leptin levels and reduces elevated leptin receptor expression in BMPR2 mutant RV cardiomyocytes.

### In cultured cardiomyocytes with BMPR2 mutation, leptin stimulation alters palmitate uptake in a mutation-specific manner

**Figure 4:**
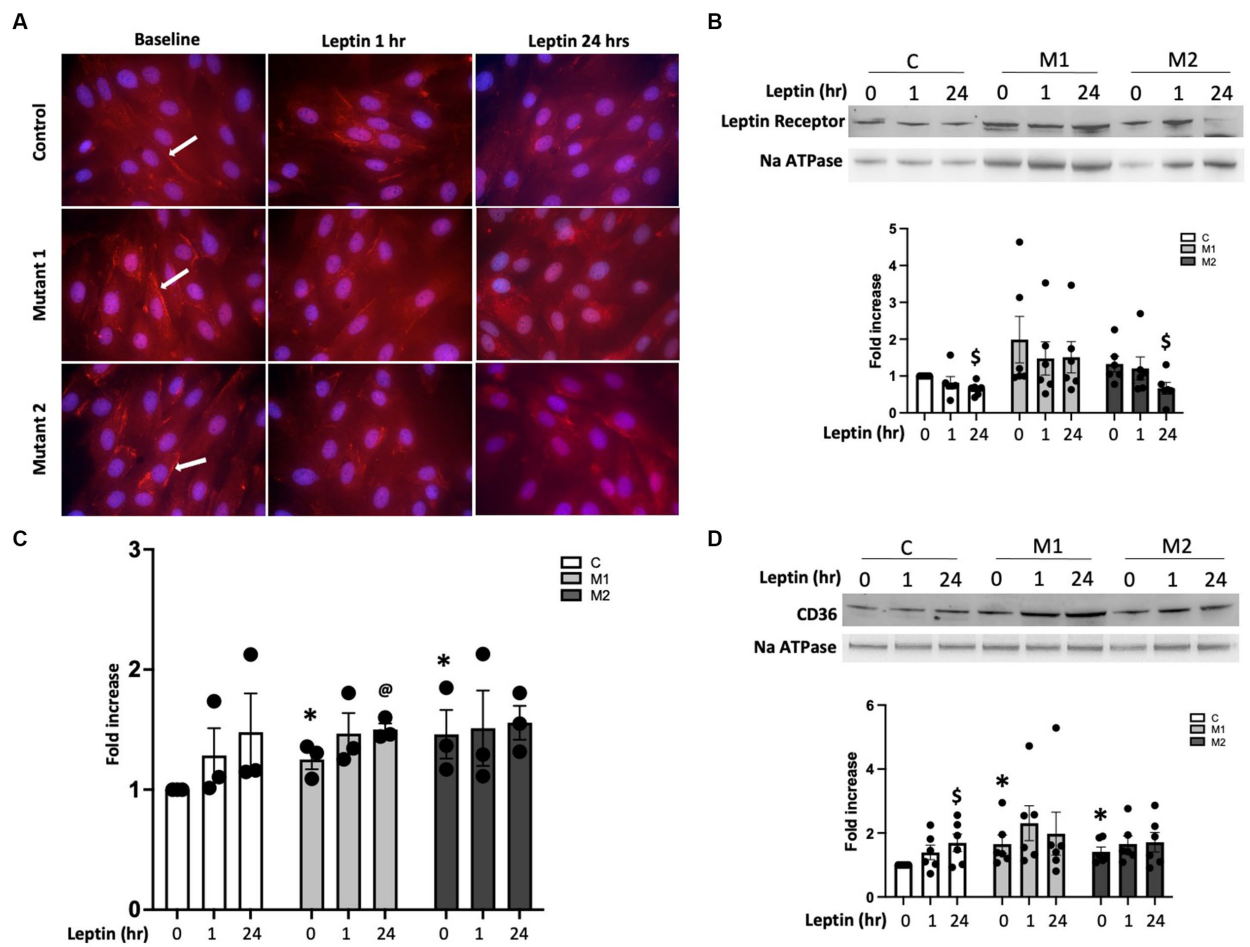
**(A)** Immunolocalization of leptin receptor in the cytoplasm and on the cell membrane (immunofluorescence staining in red, arrow depicting cell membrane localization of leptin receptor) in cultured cardiomyocytes from control, mutant 1 (M1), and mutant 2 (M2) cells at baseline and following leptin treatment for one and 24 h. Magnification 600×. **(B)** Western blot gels and bar graphs representing leptin receptor protein in cell membrane fraction in cultured cardiomyocytes from mutant and control cells at baseline and following leptin treatment (*n* = 6). ^$^*p* < 0.05, protein expression in control and M2 cells at baseline and 24 h of leptin treatment. **(C)** [^14^C] palmitate uptake in cultured cardiomyocytes from control and mutant cells following leptin treatment for 1 and 24 h (*n* = 6). ^*^p < 0.01, fold increase in palmitate uptake in mutant cells compared with control. ^@^*p* = 0.06, fold increase in palmitate uptake in mutant cells following 24 h of leptin treatment. **(D)** Western blot gels and bar graphs representing CD36 protein in cell membrane fraction in mutant and control at baseline and following leptin treatment (*n* = 6). ^$^*p* < 0.05, protein expression in control cells at baseline and 24 h leptin treatment. ^*^p < 0.05, protein expression in M1 vs. control cells and M2 vs. control cells. The statistical test used was a *t*-test.

In PAH, lipid accumulation in RV cardiomyocytes was previously published by our group and others (22, 28, 35). Leptin exposure can increase the delivery of free fatty acid substrates to left ventricular cardiomyocytes, which can lead to lipid accumulation and cardiac lipotoxicity (41, 42). It is unknown if leptin may, in part, mediate increased lipid uptake and accumulation in the PAH-RV. We, therefore, sought to understand the role of leptin and leptin receptors in free fatty acid uptake in PAH-RV cardiomyocytes using cultured cardiomyocytes (H9C2) with two different BMPR2 mutations: M1, a cytoplasmic tail domain mutation, and M2, a kinase domain mutation (Figure 4). In control and BMPR2 mutant cultured cardiomyocytes, the leptin receptor was localized in the cytoplasm as well as on the cell membrane. In the BMPR2 mutant cells, the localization of the leptin receptor appeared to be more intense (bright red immunofluorescent staining, as indicated by a white arrow) on the cell membrane compared to controls. Leptin stimulation for 1 and 24 h did not change the intensity or localization of leptin receptors in either control or BMPR2 mutant cells (Figure 4A). We, therefore, sought to define the sub-cellular localization of leptin receptors in mutant cells by enriching the cell membrane fraction. At baseline, there was no significant difference in the abundance of leptin receptors in the cell membrane fraction between mutant cells and controls (Figure 4B). While 1 h of leptin stimulation did not affect leptin receptor protein in the plasma membrane in either mutant or control cells, 24 h of leptin stimulation significantly decreased (*p* < 0.05) leptin receptor protein in control and M2 cells compared to baseline. In contrast, M1 cells had no change in leptin receptor abundance in the plasma membrane fraction despite 24 h of leptin exposure, suggesting that in the presence of this specific mutation, leptin does not downregulate cell membrane localization of leptin receptor. Next, we tested the effect of leptin on fatty acid uptake in these cultured cardiomyocytes with BMPR2 mutation using radioactive palmitate. As previously published (28), at baseline, there was a significant increase in palmitate uptake in cultured cardiomyocytes with BMPR2 mutation (Figure 4C). Consistent with leptin receptor data (Figure 4B), 1 h leptin stimulation did not show any effect on palmitate uptake in control or mutant cells, but leptin stimulation for 24 h demonstrated a trend toward (*p* = 0.06) an increase in palmitate uptake in M1 mutant cells (Figure 4C) but not M2 cells. We further explored the effect of leptin on the localization of CD36, a key fatty acid transporter molecule, at the cell membrane in mutants and controls (Figure 4D). At baseline, membrane localization of CD36 was significantly increased (*p* < 0.05) in both BMPR2 mutant cell lines compared to controls. In control cells, CD36 expression increased numerically (*p* = 0.05) 24 h after leptin stimulation but remained unchanged in both mutant cells following leptin stimulation for 1 and 24 h (Figure 4D). These data suggest that in cultured cardiomyocytes, leptin stimulation has a BMPR2 mutation-specific effect. M1, a cytoplasmic tail domain mutation, appears to ineffectively downregulate leptin receptors after 24 h of leptin stimulation coupled with a mild increase in palmitate uptake, which could potentiate lipid accumulation in a mutation-specific manner. Increased CD36 membrane localization likely does not mediate the M1 effects of leptin stimulation.

### Mutation-specific effect of leptin and metformin exposure in BMPR2 mutant cardiomyocytes

**Figure 5:**
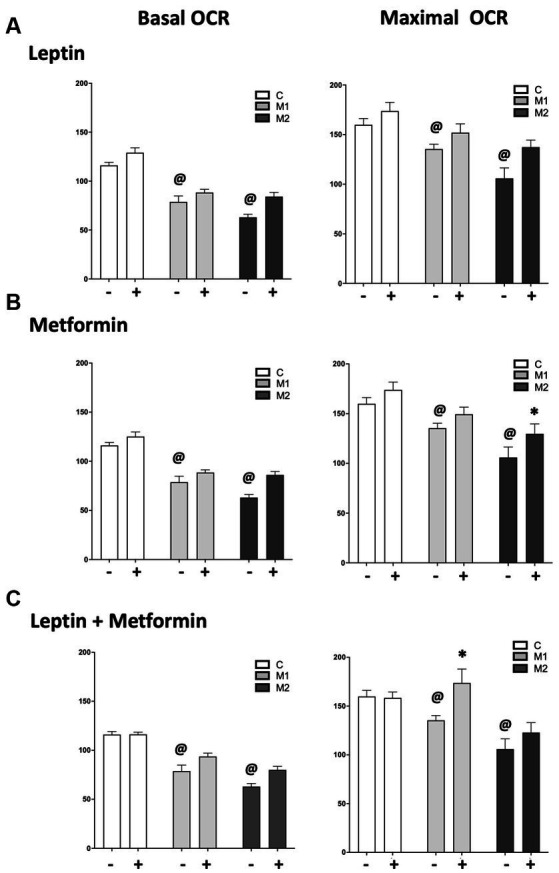
Oxygen consumption rate (OCR) measured following the addition of inhibitors of electron transport chain in the presence of palmitate in cultured cardiomyocytes from control and mutant cells grown in fatty acid oxidation (FAO) media, FAO media with leptin (6 ng/mL), FAO media with metformin (0.1 mM/mL) and FAO media with leptin (6 ng/mL) and metformin (0.1 mM/mL). **(A)** Basal and maximal OCR in control and mutant cells at baseline and following leptin treatment for 1 h. **(B)** Basal and maximal OCR in control and mutant cells at baseline and following metformin treatment for 1 h. *p* < 0.01 maximal OCR in M2 cells at baseline vs. metformin treatment. **(C)** Basal and maximal OCR in control and mutant cells at baseline and following leptin plus metformin treatment for 1 h. *p* < 0.01 maximal OCR in M1 cells at baseline vs. leptin plus metformin treatment. The statistical test used was a two-way ANOVA. ^@^*p* < 0.01 Change in basal and maximal OCR in mutant cells compared to controls.

Leptin is a liporegulatory hormone that controls lipid homeostasis in the heart (43) and can stimulate fatty acid oxidation (FAO) in muscle and the left ventricle (44). Metformin is also known to increase FAO in several tissues (45, 46), so we hypothesized that there may be an interaction between leptin and metformin in RV metabolism. We next tested the effect of leptin, metformin, or both on palmitate-driven mitochondrial respiration by measuring oxygen consumption rate (OCR) in cultured cardiomyocytes with and without BMPR2 mutation using the Seahorse XFe96 Extracellular Flux Analyzer (Figure 5). At baseline, as expected (28), basal and maximal OCR were significantly decreased (*p* < 0.01) in mutant cells compared to controls (Figures 5A–C). Following leptin stimulation, in control and mutant cells, there was a modest but not significant increase in basal and maximal OCR, especially in M2 cells compared to baseline. Furthermore, following leptin treatment in mutant cells, basal OCR remained significantly lower (*p* < 0.001) and maximal OCR trended lower than the controls (Supplementary Figure S2A). With metformin treatment, there was a modest (not significant) increase in basal OCR in control and mutant cells and a significant increase in maximal OCR in M2 but not M1 cells compared to controls (Figure 5B). Finally, when control cells were exposed to both leptin and metformin, there was no change in either basal or maximal OCR. While there was no significant change in basal OCR in either mutant cell line, maximal OCR was significantly increased (*p* < 0.001) in M1 cells but not M2 cells compared to controls (Figure 5C; Supplementary Figure S2C). These data show that leptin alone does not increase lipid-driven mitochondrial respiration in control or BMPR2 mutant cardiomyocytes, while metformin exposure increases maximal OCR in M2 cells selectively. Metformin and leptin exposure together in M1 cells alone increased lipid-stimulated mitochondrial respiration. These data further support a BMPR2 mutation-specific effect of metformin on mitochondrial function.

### Metformin alone or in combination with leptin influences genes involved in fatty acid synthesis and fatty acid oxidation in cultured cardiomyocytes with BMPR2 mutation, and the effect of metformin is BMPR2 mutation-specific

**Figure 6:**
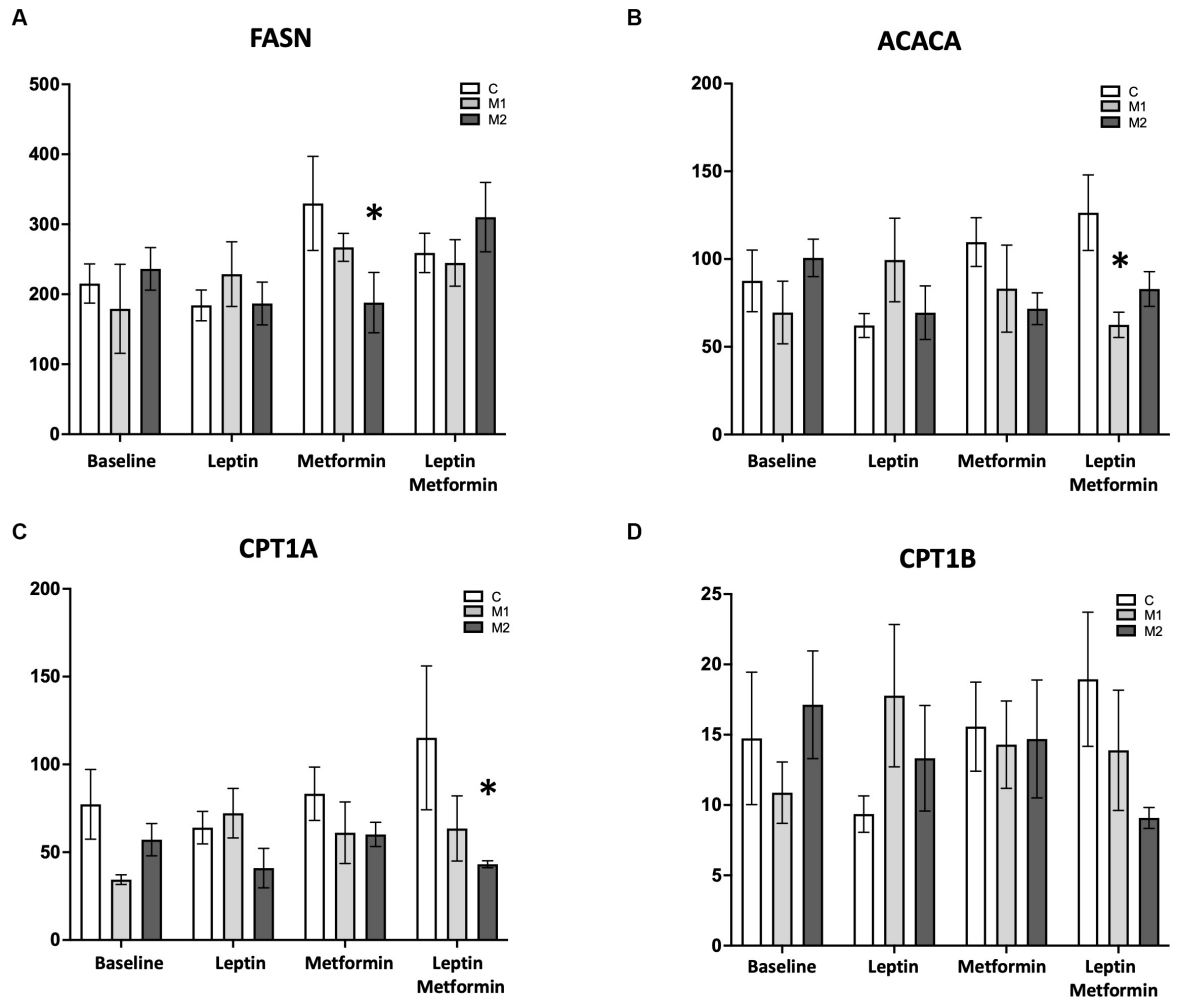
Gene expression profile in the presence of palmitate in cultured cardiomyocytes from control and mutant cells at baseline, with leptin (6 ng/mL), with metformin (0.1 mM/mL), and with leptin (6 ng/mL) and metformin (0.1 mM/mL). **(A)** FASN, **(B)** ACACA, **(C)** CPT1A, and **(D)** CPT1B ^*^p < 0.01. The statistical test used was a two-way ANOVA.

We next sought to understand potential mechanisms that may explain the BMPR2 mutation-specific metabolic findings identified in response to leptin stimulation and metabolic interventions. In cultured cardiomyocytes with two forms of BMPR2 mutation and controls, we assayed gene expression by quantitative real-time polymerase chain reaction (RT-PCR) key genes regulating lipid accumulation and mitochondrial respiration following 1 h of either leptin or metformin or both treatments. We found that leptin treatment alone does not alter genes involved in fatty acid oxidation or fatty acid synthesis in both control and mutant cells (Figures 6A–D). However, metformin alone significantly decreases (*p* < 0.05) the expression of the fatty acid synthase (FASN) gene in M2 but not M1 cells compared to control cells (Figure 6A). This gene is responsible for the synthesis of long-chain fatty acids such as palmitate from acetyl- and malonyl-CoA; thus, metformin may potentially limit fatty acid synthesis only in M2 cells. Furthermore, metformin and leptin significantly decreased (*p* < 0.01) the expression of the acetyl-CoA carboxylase gene in M1 but not M2 cells compared to controls (Figure 6B). This gene is responsible for catalyzing the carboxylation of acetyl-CoA, the rate-limiting step in fatty acid synthesis, suggesting that in the presence of leptin and metformin, acetyl-CoA may be available for fatty acid oxidation in M1 cells. In M2 cells, metformin together with leptin significantly reduced (*p* < 0.05) the expression of carnitine palmitoyltransferase 1A (CPT1A) and showed a trend toward reduction (*p* = 0.07) in the expression of the CPT1B gene compared to control cells (Figures 6C,D). Both of these genes are essential for the net transport of long-chain fatty acids from the cytoplasm to the mitochondria, suggesting acetyl-CoA may not be available for FAO in these cells. Taken together, these data indicate that metformin influences genes involved in fatty acid synthesis, and metformin, together with leptin, can influence genes involved in fatty acid oxidation in a BMPR2 mutation-specific manner.

## Discussion

In this study, we sought to understand how leptin and leptin signaling may impact the RV in PAH and whether this may be altered by a metabolic intervention that is being explored in PAH, metformin (NCT03617458). Using human samples and rodent models of the RV in PAH, we show that leptin levels in circulation and leptin receptor expression in RV cardiomyocytes are both increased in PAH compared to controls. We further show that metformin therapy reduced both plasma leptin abundance and RV leptin receptor expression. In a cardiomyocyte cell line with two different BMPR2 mutations that recapitulate metabolic features of the PAH-RV (28), we found a mutation-specific effect of leptin on metabolism. Specifically, exposure to leptin increased palmitate uptake without affecting leptin receptor expression or mitochondrial respiration in a cytoplasmic domain BMPR2 mutation, which has implications for the well-described RV steatosis in PAH. Furthermore, we observed that when cultured cardiomyocytes with BMPR2 mutation are exposed to leptin and metformin together, there is an improvement in mitochondrial respiration, suggesting that metformin may counteract the enhanced fat uptake stimulated by leptin by influencing genes involved in fatty acid oxidation, again in a BMPR2 mutation-specific manner. These data suggest that leptin may play a role in RV metabolism, and leptin-mediated RV metabolic changes can be altered by metformin in a mutation-specific manner.

There have been other publications focused on the role of leptin in pulmonary vascular disease (PAH). In accordance with prior publications in PAH patients (15, 16, 21), our data also shows an increase in the circulating levels of leptin in PAH patients and a moderately significant correlation with BMI. Similarly, our data showing increased leptin in circulation in the mouse model of PAH with BMPR2 mutation is consistent with the published data in hypoxia and the monocrotaline mouse model of PH (18), although this has never been confirmed in BMPR2 mutant mice, which have a strong recapitulation of human metabolic features (28, 47, 48). Finally, an increase in leptin receptor expression in lung tissue and circulating cells from both PAH patients and animal models of PH has previously been demonstrated (15, 19), but we, for the first time, demonstrate an increase in leptin receptor localization in PAH-RV cardiomyocytes and RV cardiomyocytes from mouse models of RV stress load. Thus, the leptin-signaling pathway may have an important role to play in the development of PAH, but these data are novel in that they suggest leptin may not just promote pulmonary vascular disease in PAH (18) but may also affect the RV in PAH.

Leptin and leptin receptors are shown to be increased more than four-fold in the failing human left ventricle (8), and in ischemic cardiac injury, leptin receptor expression is significantly increased in the RV (49). Similarly, pressure-mediated left ventricular hypertrophy and mechanical stretch also upregulate leptin receptor gene expression (39). Thus, in cardiomyocytes, the leptin receptor is an important component of the leptin-signaling pathway (39). Our study adds to this body of literature by demonstrating that in the RV of PAH patients and two mouse models of RV dysfunction, leptin receptor expression is significantly increased.

Leptin can elevate cellular lipid pools by increasing the uptake of exogenous fatty acids (42, 50). This effect is mediated via increased expression and membrane translocation of CD36, which plays a critical role in fatty acid import (44, 51). In HL-1 (a mouse cultured cardiomyocyte cell line), Palanivel et al. (42) have shown an increase in leptin-stimulated palmitate uptake trended higher compared to baseline, it is not significant. M2 mutant cells, in which the mutation is in the BMPR2 kinase domain, behaved similarly to control cells in leptin-stimulated palmitate uptake and reduction in cell surface leptin receptor. Interestingly, in M1 mutant cells with a cytoplasmic domain BMPR2 mutation, we observed a further increase in leptin-stimulated palmitate uptake, corresponding to no reduction in cell surface leptin receptor and complemented by a significant increase in leptin-stimulated palmitate uptake. In HL-1, leptin-stimulated palmitate uptake is shown to correspond with increased cell surface CD36 protein content (42, 44). Our findings in control cells show a similar increase in leptin-stimulated cell surface CD36 protein, but not in mutant cells. This could be because cell surface CD36 protein is already increased in mutant cells as a result of BMPR2 mutation. In past studies, we have demonstrated pleiotropic effects of these BMPR2 mutations in cell culture and animal studies (48, 52–54), which is consistent with the differences we observe with regard to palmitate uptake in response to leptin in these cultured BMPR2 mutant cardiomyocytes. Differences in cytoplasmic tail signaling may underlie some of our observed variability in cell culture and likely underlie the clinical heterogeneity across HPAH families.

Metformin is an antidiabetic medication with multiple potential mechanisms of action that has been shown to reduce RV lipids in PAH patients (55) and is presently under study as a therapy for PAH in a multicenter trial. In animal models of PH, metformin has been shown to prevent the deleterious effects of high fat on RV function and myocardial steatosis (22). We, therefore, sought to understand if there might be an interaction between metformin and leptin in the RV. In concordance with published findings (56–58), our data also shows that metformin reduces circulating levels of leptin in humans and in a mouse model of PH. In the PAH-RV, metformin is shown to reduce lipid deposition, which is in the form of ceramides (22), probably through inhibition of ceramide synthesis (59). In dysfunctional cardiomyocytes at the cellular level, metformin is shown to improve mitochondrial function (60) and mitochondrial biogenesis (61), promote B-oxidation of fatty acids (62), and decrease mitochondrial permeability transition pore (mPTP) opening (63, 64). Here we add the novel observation that metformin treatment also reduces RV leptin receptor protein in the PAH-RV. In cultured cardiomyocytes as well, we show a metformin-dependent improvement in maximal mitochondrial oxygen consumption rate and a decrease in FASN gene expression (a multifunctional enzyme that catalyzes the *de novo* biosynthesis of long-chain saturated fatty acids starting from acetyl-CoA and malonyl-CoA in the presence of NADPH) in the presence of available free fatty acids in BMPR2 mutation-specific manner. Metformin is also shown to enhance leptin sensitivity and correct leptin resistance in high-fat-fed obese rats, and a combination therapy including metformin and leptin is helpful in the treatment of obesity (65). In cultured cardiomyocytes, we have shown that metformin in combination with leptin can improve mitochondrial function by increasing lipid-dependent mitochondrial respiration and decreasing ACACA gene expression (the cytosolic enzyme that catalyzes the carboxylation of acetyl-CoA to malonyl-CoA, the first and rate-limiting step of *de novo* fatty acid biosynthesis) in BMPR2 mutation-specific manner, suggesting a combination of leptin and metformin may have a beneficial effect.

In conclusion, in PAH, increased circulating leptin can influence metabolic signaling in RV cardiomyocytes via leptin receptors. This pathway may play a role in altering lipid-dependent RV metabolism at the cellular level in combination with metformin in a mutation-specific manner, hence warranting further studies.

## Data availability statement

The original contributions presented in the study are included in the article/Supplementary material, further inquiries can be directed to the corresponding author.

## Ethics statement

The studies involving humans were approved by Vanderbilt University Medical Center. The studies were conducted in accordance with the local legislation and institutional requirements. The participants provided their written informed consent to participate in this study. The animal study was approved by Institutional Animal Care and Use Committee. The study was conducted in accordance with the local legislation and institutional requirements.

## Author contributions

MT: Conceptualization, Data curation, Formal analysis, Investigation, Methodology, Resources, Supervision, Writing – original draft, Writing – review & editing. EB: Funding acquisition, Writing – review & editing. VA: Funding acquisition, Methodology, Writing – review & editing. NF: Writing – review & editing. KS: Writing – review & editing. SS: Writing – review & editing. XZ: Writing – review & editing. MF: Formal analysis, Methodology, Writing – review & editing. JW: Conceptualization, Methodology, Writing – review & editing. AH: Conceptualization, Data curation, Formal analysis, Funding acquisition, Methodology, Resources, Writing – review & editing.

## Supplementary material

SUPPLEMENTARY FIGURE S1 **(A)** Plasma leptin levels in PAH patients (*n*=18) at baseline and after 8 weeks of metformin treatment. **p*<0.0001 (Wilcoxon matched-pairs signed rank test). **(B)** Correlation between plasma leptin and BMI <25 in PAH patients (*n*=9) **p*<0.0001. **(C)** Correlation between plasma leptin and BMI 25< in PAH patients (*n*=17) **p*<0.0001.

Supplementary Figure S2 Oxygen consumption rate (OCR) measured over time following the addition of inhibitors of electron transport chain in the presence of palmitate in cultured cardiomyocytes from control and mutant cells grown in fatty acid oxidation (FAO) media, **(A)** FAO media with leptin (6ng/ml), **(B)** FAO media with metformin (0.1mM/ml); *p*< 0.001 Maximal OCR in M2 cells at baseline vs metformin treatment and **(C)** FAO media with leptin (6ng/ml) and metformin (0.1mM/ml); **p*< 0.05 Maximal OCR in M1 cells at baseline vs leptin plus metformin treatment. Control cells at baseline: Black circles; Control cells with leptin: Black squares; Control cells with metformin: Black triangles; Control cells with leptin and metformin: black diamonds; M1 cells at baseline: Blue circles; M1 cells with leptin: Blue squares; M1 cells with metformin: Blue triangles; M1 cells with leptin and metformin: Blue diamonds; M2 cells at baseline: Red circles; M2 cells with leptin: Red squares; M2 cells with metformin: Red triangles; M2 cells with leptin and metformin: Red diamonds. ^@^*p*< 0.01 Basal OCR in control vs M1 and M2 cells in leptin and metformin treated cells. ^$^*p*< 0.05 Maximal OCR in control vs M2 cells in metformin treated cells. ^%^*p*< 0.001 Maximal OCR in M1 vs M2 cells in leptin plus metformin treated cells.
