# Supplementary figures and images for: A potential adverse role for leptin and cardiac leptin receptor in the right ventricle in pulmonary arterial hypertension: effect of metformin is BMPR2 mutation-specific
Source: Front Med (Lausanne). 2023 Oct 5;10:1276422. doi: 10.3389/fmed.2023.1276422 (PMC10586504; doi:10.3389/fmed.2023.1276422)

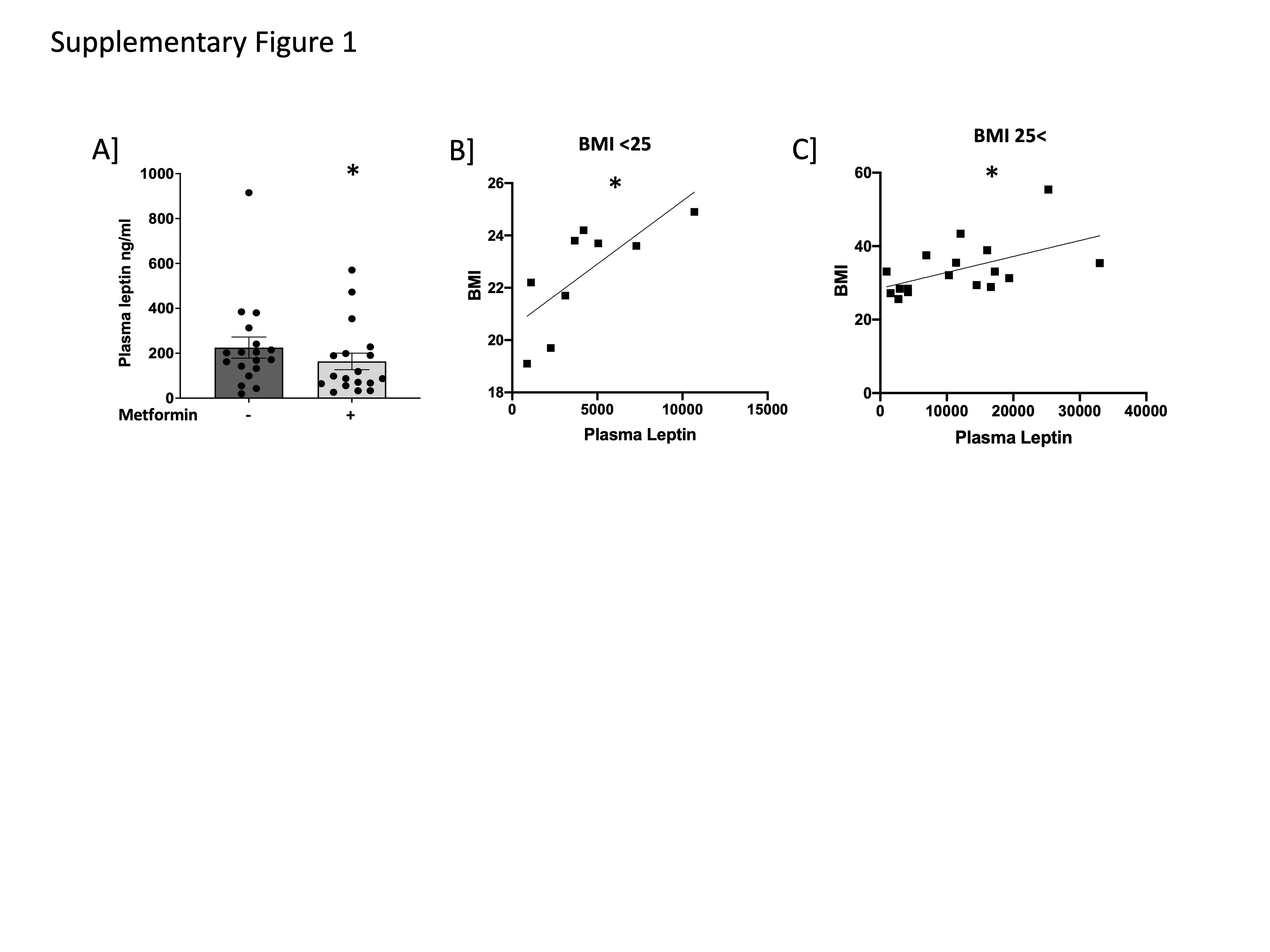

Supplement: SUPPLEMENTARY FIGURE S1 — (A) Plasma leptin levels in PAH patients (n=18) at baseline and after 8 weeks of metformin treatment. *p<0.0001 (Wilcoxon matched-pairs signed rank test). (B) Correlation between plasma leptin and BMI <25 in PAH patients (n=9) *p<0.0001. (C) Correlation between plasma leptin and BMI 25< in PAH patients (n=17) *p<0.0001. [file Image_1.JPEG]

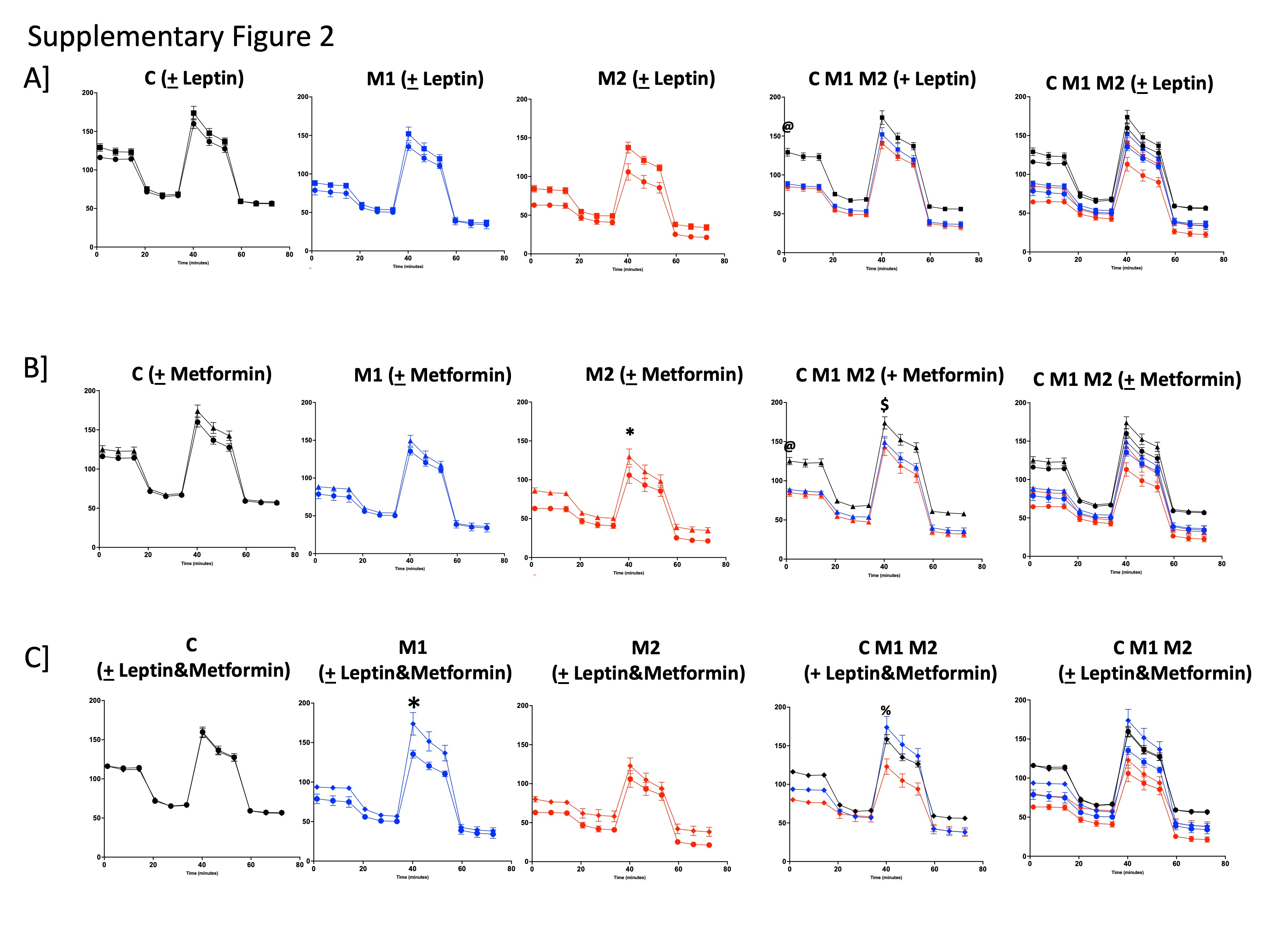

Supplement: Supplementary Figure S2 — Oxygen consumption rate (OCR) measured over time following the addition of inhibitors of electron transport chain in the presence of palmitate in cultured cardiomyocytes from control and mutant cells grown in fatty acid oxidation (FAO) media, (A) FAO media with leptin (6ng/ml), (B) FAO media with metformin (0.1mM/ml); p< 0.001 Maximal OCR in M2 cells at baseline vs metformin treatment and (C) FAO media with leptin (6ng/ml) and metformin (0.1mM/ml); *p< 0.05 Maximal OCR in M1 cells at baseline vs leptin plus metformin treatment. Control cells at baseline: Black circles; Control cells with leptin: Black squares; Control cells with metformin: Black triangles; Control cells with leptin and metformin: black diamonds; M1 cells at baseline: Blue circles; M1 cells with leptin: Blue squares; M1 cells with metformin: Blue triangles; M1 cells with leptin and metformin: Blue diamonds; M2 cells at baseline: Red circles; M2 cells with leptin: Red squares; M2 cells with metformin: Red triangles; M2 cells with leptin and metformin: Red diamonds. @p< 0.01 Basal OCR in control vs M1 and M2 cells in leptin and metformin treated cells. $p< 0.05 Maximal OCR in control vs M2 cells in metformin treated cells. %p< 0.001 Maximal OCR in M1 vs M2 cells in leptin plus metformin treated cells. [file Image_2.JPEG]
